# Esaxerenone inhibits the macrophage-to-myofibroblast transition through mineralocorticoid receptor/TGF-β1 pathway in mice induced with aldosterone

**DOI:** 10.3389/fimmu.2022.948658

**Published:** 2022-09-06

**Authors:** Panpan Qiang, Juan Hao, Fan Yang, Yutong Han, Yi Chang, Yunqian Xian, Yunzhao Xiong, Xiaomeng Gao, Lijuan Liang, Tatsuo Shimosawa, Qingyou Xu

**Affiliations:** ^1^ Graduate School, Hebei University of Chinese Medicine, Shijiazhuang, China; ^2^ Hebei Key Laboratory of Integrative Medicine on Liver-Kidney Patterns, Hebei University of Chinese Medicine, Shijiazhuang, China; ^3^ Institute of Integrative Medicine, College of Integrative Medicine, Hebei University of Chinese Medicine, Shijiazhuang, China; ^4^ Department of Clinical Laboratory, School of Medicine, International University of Health and Welfare, Narita, Japan

**Keywords:** Renal fibrosis, aldosterone, mineralocorticoid receptor blocker, macrophage-to-myofibroblast transition (MMT), M1/M2 macrophage, TGF-β1

## Abstract

Renal fibrosis is the inevitable pathway of the progression of chronic kidney disease to end-stage renal disease, which manifests as progressive glomerulosclerosis and renal interstitial fibrosis. In a previous study, we observed severe interstitial fibrosis in the contralateral kidneys of 6-month unilateral ureteral obstruction (UUO) rats, which was accompanied by increased macrophage infiltration and phenotypic transformation; after eplerenone administration, these effects were reduced. Therefore, we hypothesized that this effect was closely related to mineralocorticoid receptor (MR) activation induced by the increased aldosterone (ALD) level. In this study, we used uninephrectomy plus continuous aldosterone infusion in mice to observe whether aldosterone induced macrophage-to-myofibroblast transition (MMT) and renal fibrosis and investigated the signaling pathways. Notably, aldosterone induced predominantly M1 macrophage-to-myofibroblast transition by activating MR and upregulating TGF-β1 expression, which promoted renal fibrosis. These effects were antagonized by the MR blocker esaxerenone. These findings suggest that targeting the MR/TGF-β1 pathway may be an effective therapeutic strategy for renal fibrosis.

## 1 Introduction

Chronic kidney disease (CKD) has been considered a global public health problem with increased incidence and prevalence due to demographic expansion and major changes in epidemiologic trends, resulting in health and financial burdens (1). Renal fibrosis is the final pathological manifestation of end-stage renal disease (ESRD) and CKD (2). Renal fibrosis represents the unsuccessful healing of kidney tissue after chronic and sustained injury and is characterized by glomerulosclerosis, tubular atrophy, and interstitial fibrosis (2). Renal interstitial fibrosis (RIF) mainly manifests as a substantial accumulation of myofibroblasts and extracellular matrix (ECM) in the renal interstitial.

Myofibroblasts, which are characterized by the expression of α-SMA, are the main cells responsible for pathogenic collagen production and the predominant effector cells during tissue fibrosis (3, 4). The origin of myofibroblasts in renal fibrosis is controversial, and several cellular sources have been identified, including bone marrow-derived fibroblasts, tubular epithelial cells, endothelial cells, pericytes, and interstitial fibroblasts (3). Recently, accumulating evidence has indicated that macrophage-to-myofibroblast transition (MMT), which is a newly discovered type of cell transformation, is another possible origin of tissue myofibroblasts. Cell lineage analysis showed that MMT may be important in renal fibrosis and may be a new therapeutic target for CKD (5, 6).

Aldosterone is associated with inflammation, fibrosis, vascular damage, and end-stage organ failure (7). *In vivo* and *in vitro* results suggest the detrimental roles of aldosterone (ALD) and its receptor, which contribute to the development of renal injury and fibrosis (8). In a previous study, we observed severe interstitial fibrosis in the contralateral kidney in a 6-month unilateral ureteral obstruction (UUO) rat model, which was accompanied by increased macrophage infiltration and phenotypic transformation; after treatment with the mineralocorticoid receptor (MR) antagonist eplerenone, these effects were reduced (9). In particular, our group reported elevated plasma levels of aldosterone in UUO animals (10). Therefore, we hypothesized that this effect was closely related to MR activation induced by the increased aldosterone level.

In this study, there were three questions to be answered. 1) Can aldosterone induce MMT? 2) Which subtype of macrophage is transformed? 3) How does aldosterone work? Here we used uninephrectomy (UNX) plus continuous aldosterone infusion mouse model to examine the role of MR in MMT and RIF and provide new insights into the mechanisms of aldosterone-induced chronic renal fibrosis.

## 2 Results

### 2.1 Esaxerenone reversed hypertension and renal fibrosis in mice treated with aldosterone

**Figure 1:**
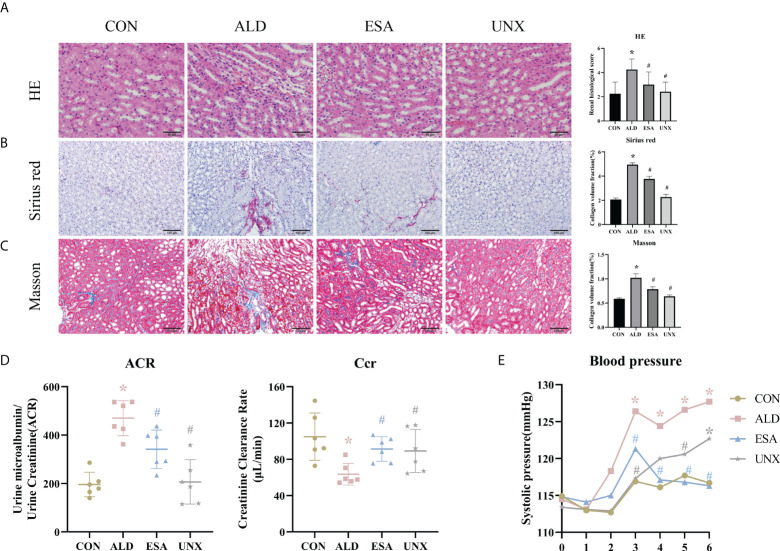
Changes in renal histology, fibrosis, and kidney function were reversed by ESA in UNX+ALD-infused mice. Kidney sections from the groups were stained with H&E to examine morphological changes and inflammatory cell infiltration **(A)**, Sirius red was used to examine collagen deposition **(B)**, and Masson was used to examine fibrosis **(C)** (n = 6). **(D)** ACR and Ccr were evaluated to determine renal function (n = 6). **(E)** Measurement of SBP (n = 10). The data are presented as the mean ± SD, ^*^p < 0.05 vs. the CON group, ^#^p < 0.05 vs. the ALD group. ESA, esaxerenone; UNX, uninephrectomy; ALD, aldosterone; ACR, urine microalbumin to urinary creatinine ratio; Ccr, creatinine clearance; SBP, systolic blood pressure.

Aldosterone-induced organ injuries were associated with inflammation. We confirmed these changes in mice with uninephrectomy and continuous aldosterone infusion with a mini-osmotic pump. As shown in **Figure 1A**, increased inflammatory cell infiltration and renal tubular damage were observed in the ALD group. Masson and Sirius red staining demonstrated that there was more collagen deposition in the ALD group than in the CON group and UNX group; additionally, no apparent difference was observed between the CON and UNX groups (**Figures 1A–C**). To determine whether aldosterone could cause kidney injury, we calculated the endogenous creatinine clearance (Ccr) rate and urine microalbumin to urinary creatinine ratio (ACR) to determine kidney function in mice. As shown in **Figure 1D**, the ACR level in the ALD group was higher and the Ccr was lower than that in the CON group. Similarly, there was no significant difference in the ACR and Ccr between the CON group and the UNX group. These effects were inhibited by a specific MR blocker (11, 12) in the esaxerenone-treated (ESA) group (**Figures 1A–D**). The systolic pressure in the ALD group mice started to increase in the third week, which was significantly different from that in the CON group, while esaxerenone antagonized the increase in systolic pressure, and the systolic pressure in the UNX group only increased in the sixth week compared with that in the CON group (**Figure 1E**). These data suggested that aldosterone could induce high blood pressure, renal injury and fibrosis, and these effects were reversed by esaxerenone.

### 2.2 Esaxerenone inhibited the aldosterone-induced infiltration of macrophages in the kidney

**Figure 2:**
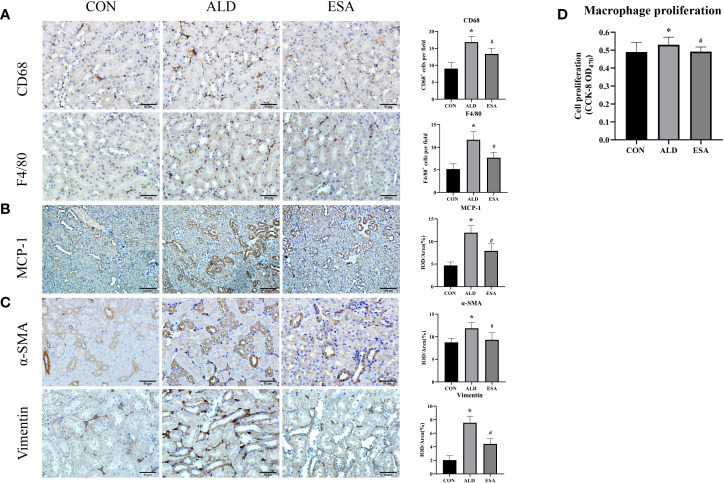
ESA antagonized the increased infiltration of macrophages and myofibroblasts. **(A)** Immunohistochemical staining using antibodies against CD68 and F4/80 to examine renal infiltration of macrophages (n = 6). **(B)** Immunohistochemical staining using antibodies against MCP-1 to examine the migration and infiltration of macrophages (n = 6). **(C)** Immunohistochemical staining using antibodies against α-SMA and vimentin to examine renal infiltration of myofibroblasts (n = 6). **(D)** CCK8 assays measured ALD-induced proliferation of RAW264.7 cells (n = 12). The data are presented as the mean ± SD, ^*^p < 0.05 vs. the CON group, ^#^p < 0.05 vs. the ALD group. ESA, esaxerenone; CCK8, Cell Counting Kit-8; ALD, aldosterone.

We used F4/80 and CD68 to identify macrophages and observed significant macrophage infiltration in the renal interstitium in the ALD group and that esaxerenone inhibited macrophage infiltration (**Figure 2A**). To investigate the reasons for this increase in macrophages, we first measured MCP-1 expression, which was upregulated in the ALD group, suggesting that ALD may promote macrophage migration and accumulation in the renal interstitium *via* MCP-1 (**Figure 2B**). In addition, we examined proliferative cells by staining mouse kidneys with Ki-67. The results showed increased positive expression of Ki-67 in renal tubular cells and renal interstitial inflammatory cells in the kidneys from the ALD group compared with the CON group (**Supplementary Figure 1**). Then, we observed macrophage proliferation in mouse kidneys by immunofluorescence costaining with the macrophage marker F4/80 and the proliferation marker Ki-67 and found several Ki-67^+^ macrophages (F4/80^+^-Ki-67^+^ cells) in the ALD group (**Supplementary Figure 2**). Furthermore, to avoid the impact on blood pressure, we used RAW264.7 cells and examined cell proliferation. We used a Cell Counting Kit-8 (CCK8) assay and found that macrophage proliferation in the ALD group was significantly higher than that in the CON group, which suggested that ALD-induced macrophage proliferation may be one of the reasons for the accumulation of macrophages in the renal interstitium *in vivo* (**Figure 2D**). The ESA group exhibited reduced MCP-1 expression *in vivo* and macrophage proliferation *in vitro*. These results suggested that MR activation increased macrophage infiltration by upregulating MCP-1 expression and promoting macrophage proliferation.

### 2.3 Esaxerenone alleviated aldosterone-induced macrophage-to-myofibroblast transition *in vivo* and *in vitro*

Myofibroblasts were evaluated by immunohistochemical staining with antibodies against α-SMA and vimentin, which are specific markers used to identify myofibroblasts. Myofibroblasts accumulated in the renal interstitium in the ALD group, and this accumulation was reversed in the ESA group (**Figure 2C**).

**Figure 3:**
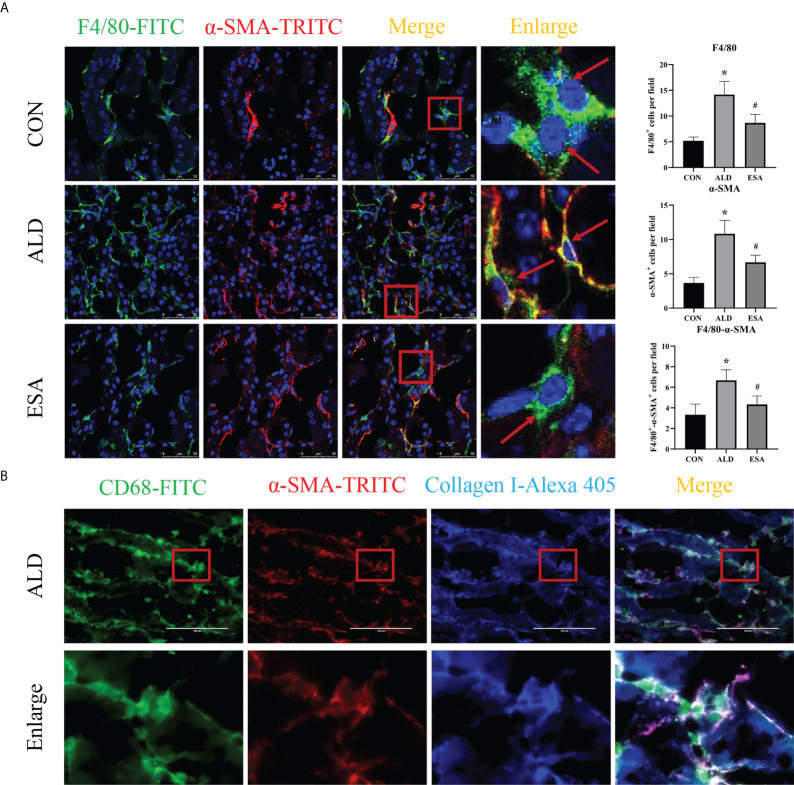
MMT in UNX+ALD-infused mice. **(A)** Immunofluorescence staining of kidney sections with antibodies against the macrophage marker F4/80 (FITC, green) and the myofibroblast marker α-SMA (TRITC, red) to identify MMT (cells co-expressing both markers indicate MMT; nuclei were stained with DAPI in blue). **(B)** Immunofluorescence staining of kidney sections with antibodies against CD68 (green), α-SMA (red), and collagen I (blue). The data are presented as the mean ± SD (n = 6), ^*^p < 0.05 vs. the CON group, ^#^p < 0.05 vs. the ALD group. MMT, macrophage-to-myofibroblast transition; UNX, uninephrectomy; ALD, aldosterone; FITC, fluorescein isothiocyanate.

**Figure 4:**
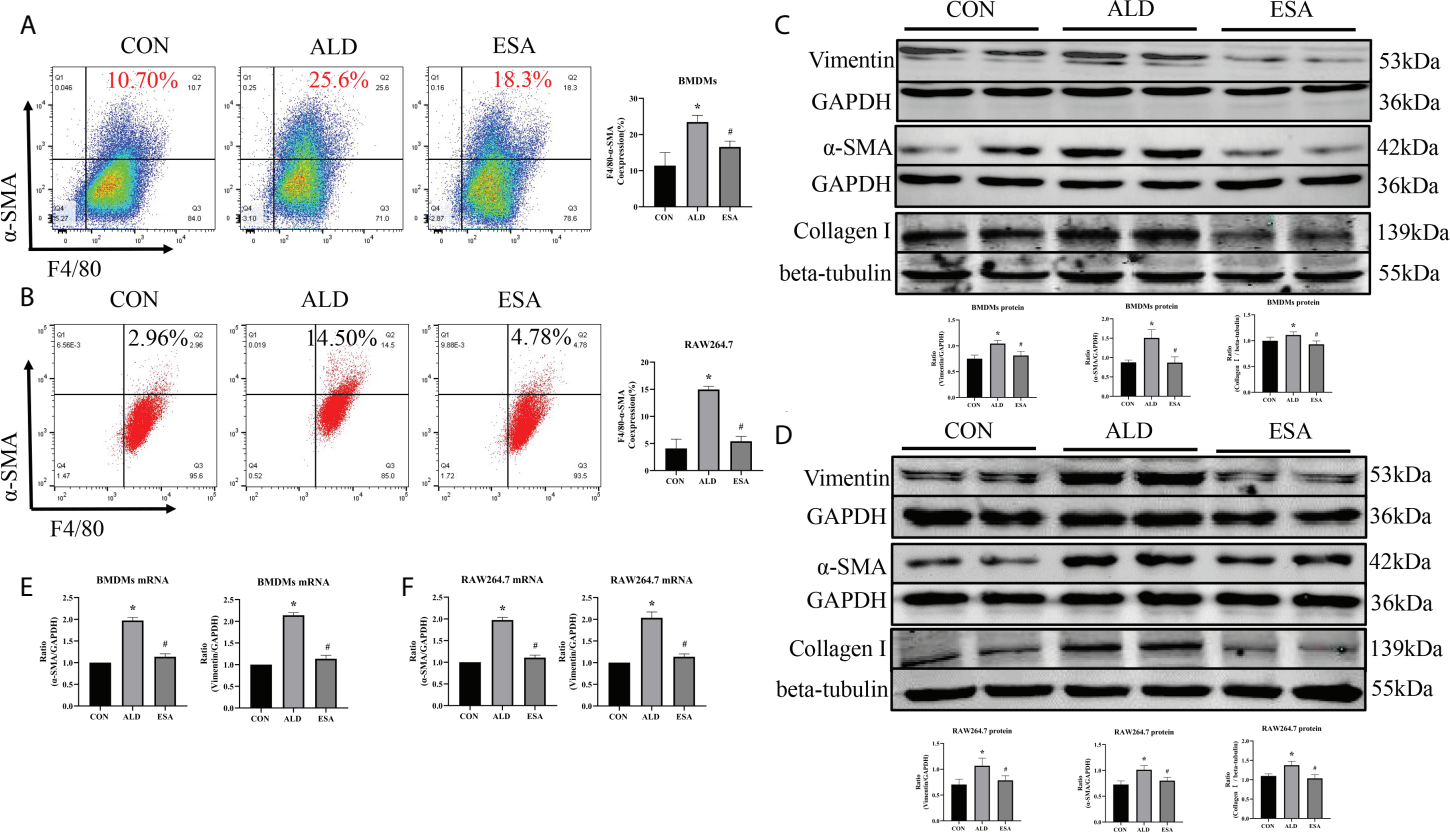
ESA antagonized MMT in ALD-treated BMDMs and RAW264.7 cells. **(A)** Flow cytometric analysis of the expression of α-SMA in BMDMs, and Q2 shows the percentage of MMT cells that were α-SMA^+^ and F4/80^+^ (n = 3). **(B)** Flow cytometric analysis of the expression of α-SMA in RAW264.7 cells, and Q2 indicates the percentage of MMT cells that were α-SMA^+^ and F4/80^+^ (n = 3). **(C)** Western blotting analysis of α-SMA, vimentin, and collagen I in BMDMs (n = 6). **(D)** Western blotting analysis of α-SMA, vimentin, and collagen I in RAW264.7 cells (n = 6). **(E)** The mRNA expression of α-SMA and vimentin in BMDMs (n = 6). **(F)** The mRNA expression of α-SMA and vimentin in RAW264.7 cells (n = 6). The data are presented as the mean ± SD, ^*^p < 0.05 vs. the CON group, ^#^p < 0.05 vs. the ALD group. ESA, esaxerenone; MMT, macrophage-to-myofibroblast transition; ALD, aldosterone; BMDMs, bone marrow-derived monocytes/macrophages.

MMT was evaluated by costaining the kidney with antibodies against α-SMA and the macrophage marker F4/80. As expected, the number of α-SMA and F4/80 double-positive cells in the ALD group was higher than that in the CON group, and this effect was reduced in the ESA group (**Figure 3A**). Furthermore, we costained CD68 and α-SMA with collagen I and found that cells expressing both CD68 and α-SMA also expressed collagen I, which suggested that the phenotypically transformed macrophages secreted collagen components and participated in fibrosis (**Figure 3B**). The same result was found in the extracted RAW264.7 and bone marrow-derived monocytes/macrophages (BMDMs) protein *in vitro*. The expression of collagen I in the ALD group was higher than that in the CON group, and it was inhibited by ESA (**Figures 4C, D**).

Subsequently, the *in vivo* findings were confirmed in an *in vitro* experiment with BMDMs and RAW264.7 cells treated with ALD. The results of two-color flow cytometry showed that the co-expression of F4/80 and α-SMA in the ALD group was significantly higher than that in the CON group and that esaxerenone inhibited the expression of α-SMA (**Figures 4A, B**). Accordingly, we extracted protein and mRNA from RAW264.7 cells and BMDMs treated with ALD and esaxerenone and found that the expression of α-SMA and vimentin in the ALD group was upregulated, and this effect was reversed in the ESA group (**Figures 4C–F**).

**Figure 5:**
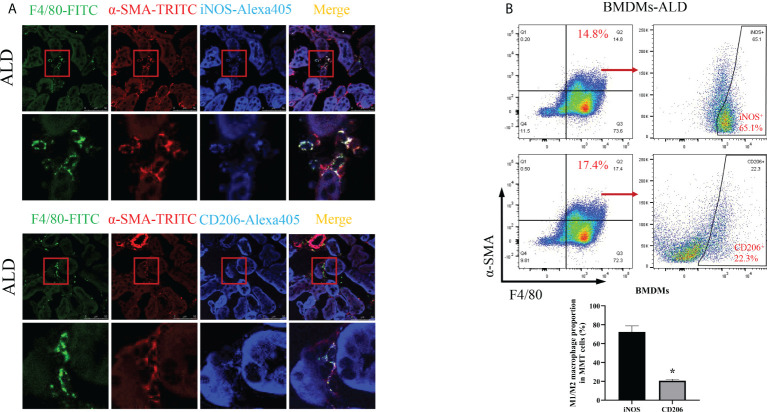
M1 macrophages were the major subtype associated with MMT in the kidneys of UNX+ALD-infused mice and ALD-treated BMDMs. **(A)** Fluorescence costaining of F4/80 (green) and α-SMA (red) with the M1 macrophage marker iNOS (blue) or the M2 macrophage marker CD206 (blue). **(B)** Flow cytometric analysis of the expression of F4/80, α-SMA, and iNOS or CD206 in ALD-treated BMDMs. The data are presented as the mean ± SD, *p < 0.05 vs. iNOS. MMT, macrophage-to-myofibroblast transition; UNX, uninephrectomy; ALD, aldosterone; BMDMs, bone marrow-derived monocytes/macrophages.

To further investigate which subtype of macrophage was the main participant in MMT, we conducted the following experiments. First, we performed an immunohistological study by costaining kidney sections with F4/80, α-SMA, and iNOS (an M1 macrophage marker) or CD206 (an M2 macrophage marker) and found that the expression of iNOS was higher than that of CD206 in MMT cells (**Figure 5A**). In addition, BMDMs were examined by three-color flow cytometry. After typical gating, MMT cells were identified by the expression of F4/80 and α-SMA, and then the ratio of M1 and M2 cells was determined in the F4/80^+^α-SMA^+^ population. After stimulation with ALD, the proportion of M1 macrophages was 72.33% ± 6.43%, and that of M2 macrophages was 20.60% ± 1.75% among BMDMs (**Figure 5B**). These results indicated that F4/80^+^α-SMA^+^ cells expressed increased iNOS, suggesting that M1 macrophages were transformed into myofibroblasts.

### 2.4 Aldosterone induced renal fibrosis and macrophage-to-myofibroblast transition by activating mineralocorticoid receptor *in vitro*

**Figure 6:**
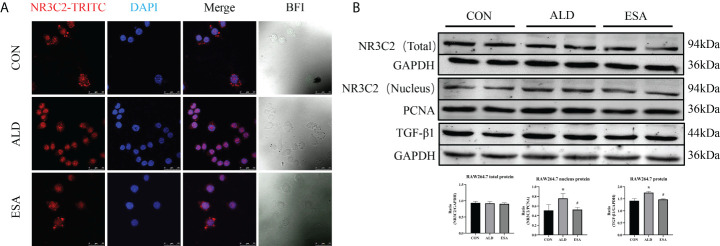
Effects of ALD and ESA on the expression of MR and its downstream molecule TGF-β1. **(A)** Immunofluorescence staining of kidney sections with antibodies against the MR marker NR3C2 (red) and DAPI (blue) to examine the nuclear translocation of MR. **(B)** Western blotting analysis of MR in total and nuclear protein fractions and its downstream molecule TGF-β1 in the total protein of RAW264.7 cells. The data are presented as the mean ± SD (n = 6), ^*^p < 0.05 vs. the CON group, ^#^p < 0.05 vs. the ALD group. ALD, aldosterone; ESA, esaxerenone; MR, mineralocorticoid receptor.

To investigate the role of MR in these outcomes, we first examined the localization of the MR marker NR3C2 to confirm the activation of MR. NR3C2 was mainly observed in the cytoplasm in the CON group, while in the ALD group, NR3C2 was transferred to the nucleus in RAW264.7 cells (**Figure 6A**). The Western blotting results showed that there was no difference in total MR expression among the CON, ALD, and ESA groups; however, the expression of MR in the nucleus was significantly different, and the expression of MR in the ALD group was higher than that in the CON group, while esaxerenone antagonized the nuclear accumulation of MR (**Figure 6B**).

**Figure 7:**
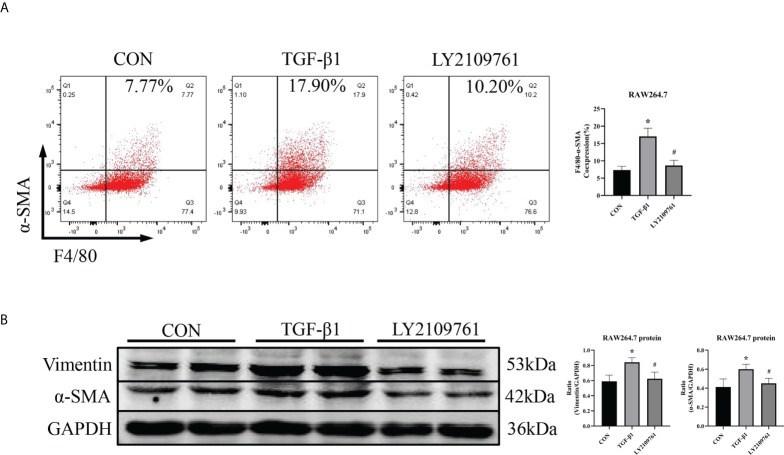
The TGF-β1 receptor blocker LY2109761 antagonized MMT in TGF-β1-treated RAW264.7 cells. **(A)** Flow cytometric analysis of the expression of α-SMA in RAW264.7 cells, and Q2 indicates the percentage of MMT cells (n = 3). **(B)** Western blotting analysis of α-SMA and vimentin expression in RAW264.7 cells stimulated with TGF-β1 and treated with or without LY2109761 (n = 6). The data are presented as the mean ± SD, ^*^p < 0.05 vs. CON, ^#^p < 0.05 vs. TGF-β1. MMT, macrophage-to-myofibroblast transition.

**Figure 8:**
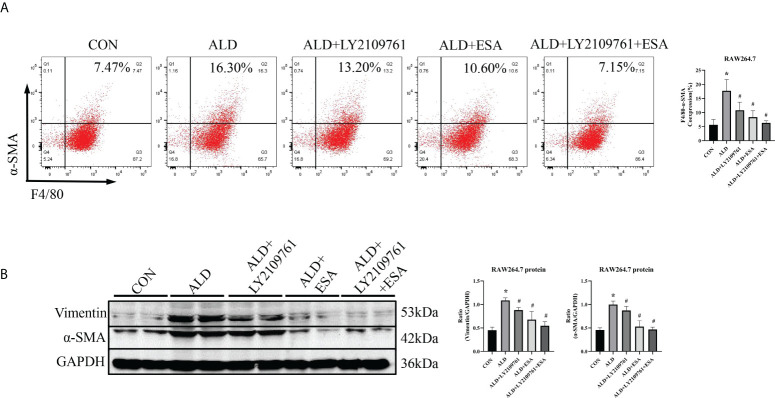
The TGF-β1 receptor blocker LY2109761 antagonized ALD-treated MMT in RAW264.7 cells. **(A)** Flow cytometric analysis of the expression of α-SMA in RAW264.7 cells, and Q2 indicates the percentage of MMT cells (n = 3). **(B)** Western blotting analysis of α-SMA and vimentin expression in ALD-treated RAW264.7 cells (n = 6). The data are presented as the mean ± SD, ^*^p < 0.05 vs. the CON group, ^#^p < 0.05 vs. the ALD group. ALD, aldosterone; MMT, macrophage-to-myofibroblast transition.

Additionally, we found increased expression of TGF-β1, one of the downstream molecules of MR, in the ALD group (**Figure 6B**). To further investigate whether TGF-β1 plays an important role in MMT, we cultured RAW264.7 cells with TGF-β1 (10 ng/ml) for 24 h and found that the expression of α-SMA was upregulated (**Figure 7A**). The addition of the TGF-β1 receptor blocker LY2109761 inhibited the transition of macrophages into myofibroblasts (**Figures 7A, B**). Moreover, the expression of α-SMA in the ALD+LY2109761 group was lower than that in the ALD group, which indicated that the TGF-β1 inhibitor could antagonize ALD-induced MMT (**Figures 8A, B**). These results suggest that the MR/TGF-β1 pathway is involved in MMT and plays a regulatory role.

## 3 Discussion

Renal fibrosis is the inevitable pathway of the progression of CKD to ESRD, which manifests as progressive glomerulosclerosis, RIF, and other pathological conditions. In previous studies, we used the 6-month rat UUO model and found an increase in plasma ALD level and fibrotic changes in the contralateral kidney, and we further reported that MMT induced renal fibrosis (9, 10, 13). We hypothesized that UUO increased plasma ALD levels, activated MR, and induced MMT to promote renal fibrosis. There is evidence that a high-salt diet and unilateral nephrectomy can accelerate ALD-induced kidney injury (14, 15). Therefore, based on previous findings, we used a 6-week uninephrectomy plus ALD-infused mouse model and cell culture and further investigated the mechanism of ALD-induced MMT in renal fibrosis. In the current study, renal function and renal pathology in the UNX group showed no significant changes compared with those in the CON group, which suggested that 6-week uninephrectomy did not cause renal injury in mice. In contrast, when UNX mice were treated with ALD, apparent renal fibrosis and kidney injury were observed. Esaxerenone reversed the effects of exogenous ALD; however, blood pressure was also reduced *in vivo* in the study. To investigate the role of MR independent of the effect on blood pressure, we treated cells with ALD and studied the activation of MR, transition of cell phenotype, and the signal transduction of MR. We showed that ALD activated MR and TGF-β1 and further induced M1 prominent macrophage transition and MMT to cause fibrotic changes in the kidney.

First, ALD induces inflammation and the production of inflammatory mediators that recruit immune cells, leading to local inflammation and characteristic tissue changes and fibrosis (7, 16). CKD, however, can be considered a state of relative aldosteronism. In human clinical studies, elevated plasma ALD levels are a risk factor for kidney injury (17). In our study, we continuously infused ALD to mimic the clinical condition of CKD patients with renal injury, and we observed changes in renal pathology, deterioration of renal function, and an increase in blood pressure in model mice.

Many studies have consistently shown that ALD can activate innate and adaptive immune cells, such as macrophages and T cells, which contribute to end-organ damage in cardiovascular and metabolic diseases (16). Macrophages have important effects on kidney injury, inflammation, and fibrosis and are pleiotropic inflammatory cells involved in inflammatory responses (18). In our study, ALD prompted increased infiltration of inflammatory macrophages in the renal interstitium, and Masson and Sirius red staining showed collagen accumulation in the ALD group; after ALD was blocked with esaxerenone, the infiltration of inflammatory cells and the degree of fibrosis were alleviated. Moreover, we found that ALD induced the high expression of MCP-1 in mice, which is one of the key chemokines that regulate the migration and infiltration of monocytes/macrophages (19) and RAW264.7 macrophage cell line proliferation. We also found a few Ki-67^+^ macrophages in the kidney of aldosterone-treated mice (**Supplementary Figures 2**, **3**). Functional macrophages in tissues or organs are generally considered to be recruited from the blood or resident in tissues. It is interesting to note that several studies have recently demonstrated *in situ* proliferation of macrophages (20–23). However, the mechanism of tissue macrophage proliferation in ALD-infused mice needs to be investigated more deeply. We believe that the degree of fibrosis is closely related to the infiltration of macrophages in the renal interstitium. Our experimental results were similar to clinical studies that showed obvious macrophage infiltration in the biopsy tissue of CKD patients, which manifested as fibrosis (24), and macrophage infiltration in renal biopsies from patients was inversely associated with interstitial fibrosis and prognosis (25).

Myofibroblasts drive tissue fibrosis, and macrophages not only secrete factors associated with the generation, survival, and proliferation of myofibroblasts (26) but also transition to myofibroblasts (5, 6). Thus, this study provided direct evidence for ALD-induced MMT *in vivo* and *in vitro*. In particular, the ratio of F4/80 and α-SMA expression in ALD-treated BMDMs and RAW264.7 cells was significantly increased. In addition, immunofluorescence triple staining with CD68, α-SMA, and collagen I *in vivo*, together with extraction of RAW264.7 and BMDM protein *in vitro*, revealed that aldosterone induced an increase in collagen I secretion. Accordingly, we suggested that some of the macrophages are involved in renal fibrosis through transformation.

Moreover, in this study, BMDMs were stimulated with ALD for 24 h, and the proportion of iNOS^+^ cells among F4/80^+^-α-SMA^+^ cells undergoing MMT was higher than that of CD206^+^ cells. These findings suggest that MMT cells, which are phenotypically transformed macrophages, were dominated by M1-type macrophages. The heterogeneity of macrophage polarization has been recognized as an important feature of renal disease (27), and these cells can initially be classified as the classically activated M1 type or alternately activated M2 type. M1 macrophages can release proinflammatory chemokines and have proinflammatory functions, while M2 macrophages are associated with immune regulation and tissue remodeling, and these cells play an important role in inhibiting inflammation and tissue repair. In previous MMT studies, the macrophages involved in phenotypic transformation were mostly the M2 type, probably because M2 macrophages produce a large amount of profibrotic factors that promote myofibroblast proliferation, survival, and activation, as well as ECM overproduction (28, 29). However, among the transformed macrophages in this study, M1 macrophages were predominant, and we hypothesized that this effect may be related to the proinflammatory effect of ALD. Following kidney injury, locally produced chemokines induce the infiltration of neutrophils and naive monocytes, which differentiate into phagocytic macrophages and then polarize into different subtypes depending on the immune microenvironment (30). Our model was treated with ALD, which increases proinflammatory factors and may promote polarization toward the proinflammatory M1 phenotype. In a previous study, we also found that MMT macrophages were predominantly the M1 type under hypoxic conditions, which might be associated with the early stages of renal injury (31). Some studies also suggest that ALD induces M1 polarization in macrophages ( (32–34).

As a downstream substrate of MR, TGF-β1 can rapidly induce profibrotic effects through mRNA and protein expression (35). TGF-β1 is produced by various cells, including epithelial cells, macrophages, and myofibroblasts, and has many cellular targets that are upregulated in all forms of CKD and fibrosis in other organs and are important drivers of ECM production (26, 36). TGF-β also induces the expression of MCP-1 in tubular epithelial cells and may promote monocyte recruitment and macrophage accumulation (37). This may be one of the reasons for macrophage accumulation in ALD-treated mice. ALD increased the mRNA expression of TGF-β1 and collagen and eventually led to fibrosis in uninephrectomied rats in the presence of AT1 receptor blockade, which suggests that ALD acts independently of angiotensin II in renal fibrosis through the TGF-β1 signaling pathway (38). Another study showed that BMDMs is an important source of α-SMA^+^ myofibroblasts that accumulate in active fibrotic lesions in experimental kidney disease through MMT. This process is mediated by the TGF-β/Smad3 signaling pathway (6). *In vitro*, ALD activated MR and then increased the expression of TGF-β1. The TGF-β1 receptor blocker LY2109761 alleviated MMT induced by both TGF-β1 and ALD, which indicated that ALD could induce MMT and participate in renal fibrosis through the TGF-β1 signaling pathway.

This experiment focuses on the role of the genomic effects of aldosterone in the phenotypic transformation of macrophages. Although we have not yet verified the role of the non-genetic effect of aldosterone in MMT, genomic and non-genomic effects interact, and it is reported that the non-genomic effects of aldosterone are involved in the polarization of macrophages through MAPK and PKC activation (39, 40). Both proinflammatory factors, such as TNF-α secreted by M1 macrophages, and profibrotic factors, such as TGF-β secreted by M2 macrophages, through autocrine or paracrine signaling may have an impact on the phenotypic transformation of macrophages, so non-genomic effects may also play a role in MMT. We also found that the serum potassium levels of the aldosterone-infused mice were lower than those of the CON group (**Supplementary Figure 3**). Hypokalemia is involved in renal macrophage infiltration (41) and promotes MR activation (42); however, the role of hypokalemia on MMT needs to be furtherly investigated.

In conclusion, we performed *in vitro* and *in vivo* experiments and showed that ALD-mediated MR activation upregulated the expression of TGF-β1 and induced the transformation of macrophages, especially M1 macrophages, to myofibroblasts to participate in renal fibrosis, and the MR blocker esaxerenone antagonized these effects. The ALD/MR/TGF-β1 signaling pathway induces MMT and is involved in renal fibrosis. These findings suggest that targeting the aldosterone pathway may be an effective therapeutic strategy for renal fibrosis.

## 4 Materials and methods

### 4.1 Animals and experimental models

All experiments were carried out in accordance with recommendations for the Care and Use of Laboratory Animals in the National Institutes of Health Guidelines. Animal care followed the criteria of the Ethics Committee on Animal Experimentation of the Hebei University of Chinese Medicine. All efforts were made to minimize pain and distress to the animals.

Forty 6- to 8-week-old male (24.65 ± 1.12 g) SPF C57BL/6 mice (Charles River, Beijing, China) were maintained with standard mouse chow and tap water at room temperature under a 12-h light/12-h dark cycle. Forty mice were randomly assigned to the CON group, UNX group (uninephrectomy), ALD group (uninephrectomy+ALD infusion with mini-osmotic pump), and ESA group (uninephrectomy+ALD infusion with mini-osmotic pump+esaxerenone) (n = 10 each). Surgery was performed after 1 week of adaptive feeding. Left nephrectomy was performed in the UNX group. ALD (CAS NO.: 52-39-1, Cayman Chemical, Ann Arbor, MI, USA) infusion with a mini-osmotic pump (0.75 μg/h, ALZET model 2006, DURECT Corporation, Cupertino, CA, USA) was performed 1 week after left nephrectomy in the ALD group and ESA group. Esaxerenone (kindly provided by Daiichi Sankyo Co., Ltd., Tokyo, Japan) was administered to the ESA group *via* diet at a dose of 1 mg/kg diet for 6 weeks, and the other groups were fed regular chow. Six weeks after surgery, all animals were euthanized, and blood and right kidney tissue samples were collected.

### 4.2 Blood pressure and biochemical parameter analysis

After surgery, systolic blood pressure (SBP) was measured weekly in conscious animals by the tail-cuff method (BP-2000, Visitech Systems, Apex, NC, USA). Two days before the end of the experiment, random urine and 24-urine samples were collected, and the urine volume was recorded. Random urine was collected to measure urine creatinine (UCr) and microalbuminuria (mALB). After 6 weeks, blood samples were drawn to measure serum creatinine (SCr). UCr and SCr were measured by commercial kits (Beckman Coulter Experiment System Co., Ltd., Suzhou, China, No. AUZ3562). mALB was measured by commercial kits (Nanjing Jiancheng Bioengineering Institute, Nanjing, China, No. E038-1-1). Based on these data, the urine microalbumin/urine creatinine ratio (ACR) and Ccr were calculated (43). Serum potassium ions level was measured by protein hydrolysis enzyme method using a potassium assay kit (Changchun Huili Biotech Co., Ltd., Changchun China, No. K060).

### 4.3 Histological analysis, immunohistochemistry, and immunofluorescence analysis

The kidneys were dehydrated with alcohol and embedded in paraffin blocks after being fixed overnight in 4% paraformaldehyde (PFA). Paraffin blocks were cut into 6-μm sections for H&E, Masson, and Sirius red staining and immunohistochemical analysis of F4/80 (1:200, Invitrogen, Carlsbad, CA, USA, Cat#: PA5-21399), CD68 (1:200, Abcam, Cambridge, UK, Cat#: ab955), α-SMA (1:200, ABclonal, Woburn, USA, Cat#: A17910), vimentin (1:200, Abcam, Cat#: ab8978), MCP-1 (1:100, Proteintech, Chicago, IL, USA, Cat#: 66272-1-Ig), and anti-Ki-67 (1:100, Abcam, Cat#: ab15580). Images were observed and imaged using a Leica BX53 optical microscope (Leica, Wetzlar, Germany).

H&E staining (inflammatory cell infiltration and tubulointerstitial changes) was semiquantitatively graded in a blinded manner by two investigators. The two items were scored as 0, 1, 2, and 3 (normal, minor, moderate, and severe, respectively), and the total score was 0–6 (9). A semiquantitative analysis of Masson staining and Sirius red staining was performed according to the percentage of the collagen-positive area. Image analyses were performed using ImageJ 6.0 software (US National Institutes of Health, Bethesda, MD, USA).

### 4.4 Immunofluorescence analysis

For fluorescence staining, kidneys were irrigated with 4% PFA, dehydrated in 30% sucrose, and frozen in OCT compound (Sakura, Torrance, CA, USA). 6µm kidney sections were cut using a freezing microtome and prepared for staining with Alexa Fluor 555-conjugated α-SMA (1:500, Abcam, Cat#: ab202509) or the following unconjugated antibodies: anti-collagen I (1:50, Abcam, Cat#: ab270993), anti-Ki-67 (1:100, Abcam, Cat#: ab15580), anti-F4/80 (1:200, Abcam, Cat#: ab186073), anti-iNOS (1:50, Novus, Cat#: NB300-605), and anti-CD206 (1:50, Abcam, Cat#: ab64693). Then the sections were subjected to second or third fluorescence staining. After being stained, sections were incubated with or without DAPI for nuclear staining and sealed for photography using a confocal microscope (CTS SP8, Leica, Germany).

### 4.5 Protein extraction and Western blotting analysis

RAW264.7 cell and BMDM lysates were extracted with radioimmunoprecipitation assay (RIPA) lysis buffer (BestBio, Shanghai, China, Cat#: BB-3201) for total protein isolation and a Nuclear Protein Extraction Kit (Solarbio, Beijing, China, Cat#: R0050) for nuclear protein isolation according to the protocol recommended by the manufacturers. Western blotting was performed using sodium dodecyl sulfate–polyacrylamide gel electrophoresis (SDS–PAGE) and polyvinylidene difluoride (PVDF) membranes. After being blocked with 5% non-fat milk, the membranes were incubated with primary antibodies against collagen I (1:50, Abcam, Cat#: ab270993), NR3C2 (1:1,000, Proteintech, Cat#: 21854-1-AP), TGF-β1 (1:1,000, Abcam, Cat#: ab215715), α-SMA (1:1,000, ABclonal, Cat#: A17910), and vimentin (1:1,000, Abcam, Cat#: ab8978) overnight at 4°C. The next day, the blots were incubated with fluorescein-conjugated secondary antibodies at 1:10,000–1:20,000 for 1 h at room temperature and scanned with a Dual Color Infrared Laser Imaging Scanner (Odyssey, LICOR, Lincoln, NE, USA). Protein expression was measured with ImageJ by quantifying the density of the target total protein relative to GAPDH (1:1,000, Proteintech, Cat#: 60004-1-lg), beta-tubulin (1:1,000, Affinity, Cat#: T0023), or the target nucleoprotein relative to proliferating cell nuclear antigen (PCNA) (1:1,000, Proteintech, Cat#: 10205-2-AP).

### 4.6 Reverse transcription and quantitative real-time PCR

Total RNA was isolated from RAW264.7 cells and BMDMs using the EZNA Total RNA Kit II (Omega, Bio-Tek, Norcross GA, USA, Cat#: R6934-01). MonScript RTIII All-in-One Mix with dsDNase (Monad Biotech Co., Ltd, Shanghai, China, Cat#: MR05101 M) was used to reverse transcribe the RNA, and real-time PCR was performed using MonAmp ChemoHS qPCR Mix (Monad Biotech Co., Ltd, Shanghai, China, Cat#: MQ00401S) on an Mx3005p real-time PCR instrument. For real-time PCR, sequence-specific primers for vimentin, α-SMA, and GAPDH were as follows: vimentin: forward 5′-GCAGTATGAAAGCGTGGCTG-3′, reverse 5′-CTCCAGGGACTCGTTAGTGC-3′; α-SMA: forward 5′-TCAGGGAGTAATGGTTGGAATG-3′, reverse 5′-CCAGAGTCCAGCACAATACCAG-3′; GAPDH: forward 5′-CCTCGTCCCGTAGACAAAATG-3′, reverse 5′-TGAGGTCAATGAAGGGGTCGT-3′. The mRNA levels of α-SMA, vimentin, and GAPDH were used as housekeeping genes for normalization and were calculated using the 2^−ΔΔCT^ method.

### 4.7 *In vitro* cell culture assays

RAW264.7 cells (Procell Life Science and Technology Co., Ltd., Wuhan, China, Cat#: CL-0190) were maintained in culture media with 10% heat-inactivated fetal bovine serum (FBS) and 1% penicillin and streptomycin at 37°C in an incubator with a humidified atmosphere and 5% CO_2_. Fresh bone marrow cells (BMDMs) were harvested from C57BL/6 mice and were maintained in 1640 culture media with 30 ng/ml of macrophage colony-stimulating factor (M-CSF) (MCE, Shanghai, China, Cat#: HY-P7085) and 10% heat-inactivated FBS and 1% penicillin and streptomycin for 7 days (half fresh media was added on day 3, and sufficient fresh media was added on day 5) in an incubator under the same conditions as RAW264.7 cells. After 7 days, the cells were used for experiments. RAW264.7 cells and BMDMs were divided into the CON group, ALD group (10^−7^ mol/L ALD was administered for 24 h), and ESA group (cells were pretreated with 10^−6^ mol/L of esaxerenone 2 h prior to ALD treatment). In some experiments, RAW264.7 cells were induced with 10 ng/ml of TGF-β1 (MCE, Shanghai, China, Cat#: HY-P70543) and treated with or without the TGF-β1 receptor blocker LY2109761 (2 × 10^−6^ mol/L) (MCE, Shanghai, China, Cat#: HY-12075), and RAW264.7 cells were treated with LY2109761 after being induced with ALD.

#### 4.7.1 Flow cytometry

Twenty-four hours after treatment, RAW264.7 cells and BMDMs were harvested and stained with fluorescein isothiocyanate (FITC)-conjugated anti-F4/80 (1:100, Invitrogen, Cat#: 11-4801-82), APC-conjugated anti-α-SMA (1:1,000, Abcam, Cat#: ab202296) and anti-CD206 (1:100, Abcam, Cat#: ab64693) or iNOS (1:100, Abcam, Cat#: ab15323) for 1 h, and then the secondary antibody goat anti-rabbit IgG H&L (PE) was preabsorbed (1:500, Abcam, Cat#: ab72465) for 1 h in the dark. Unstained cells were used as negative controls. Cells were analyzed on a BD FACSAria II flow cytometer (BD Biosciences, Franklin Lake, NJ, USA), viable singlet cells were selected by FSC/SSC gating, and data were further analyzed by FlowJo 10 software.

#### 4.7.2 Cell counting kit-8

A CCK8 (MCE, Shanghai, China, Cat#: HY-K0301) assay was conducted in accordance with the manufacturer’s instructions. In brief, RAW264.7 cells were seeded at a density of 10^6^ cells/well in a 96-well plate and divided into the CON, ALD, and ESA groups. Twenty-four hours after treatment, 10 μl of CCK8 solution was added to each well and incubated for 2 h. The absorbance at 470 nm was evaluated using a VersaMax Microplate reader (Molecular Devices, Sunnyvale, CA, USA).

#### 4.7.3 Immunofluorescence cell staining

The dried and sterilized glass slides were placed on a 24-well petri dish, the cells were spread evenly on the glass slides in the wells, and 4% PFA was added and incubated for 20 min at room temperature. Then 0.25% Triton X-100 was added and incubated for 15 min for permeabilization, and 10% normal goat serum was added and incubated for 30 min for blocking. Subsequently, the cells were incubated with the primary antibody NR3C2 (1:100, Abcam, Cat#: ab64457) overnight at 4°C, followed by the relevant secondary antibody at 37°C in the dark for 1 h for fluorescence staining. DAPI was used for nuclear staining. If needed, the other antibodies were incubated for multiple staining.

### 4.8 Statistical analysis

Statistical analysis was performed using SPSS version 24.0 software (IBM, Armonk, NY, USA). All data were analyzed using a one-way analysis of variance (ANOVA) followed by the least significant difference (LSD) test for multiple groups and the independent sample t-test for two groups. The data are expressed as the mean ± SD, and a p-value <0.05 was considered statistically significant.

## Data availability statement

The raw data supporting the conclusions of this article will be made available by the authors, without undue reservation.

## Ethics statement

This study was reviewed and approved by Ethics Committee of Hebei University of Chinese Medicine.

## Author contributions

PQ performed the *in vivo* experiments and analyzed the data. JH and FY performed the *in vitro* experiments and analyzed the data. YH and YC helped with maintaining the lab animals and measuring the blood pressure of mice. YQX and YZX helped with maintaining the RAW264.7 cells and bone marrow-derived macrophages. XG and LL helped with the imaging and analysis. QX and TS designed the experiments and revised the manuscript. PQ wrote the manuscript. All authors contributed to the article and approved the submitted version.

## Acknowledgments

We thank the Hebei Key Laboratory of Integrative Medicine on Liver-Kidney Patterns of the Hebei University of Chinese Medicine. Esaxerenone was provided by Daiichi Sankyo Co., Ltd. This research was supported by the National Natural Science Foundation Project of China (QX, 81873251, 82174317) and the Construction Program of new research and development platform and institution, Hebei Province Innovation Ability Promotion Plan (No. 20567624H).

## Conflict of interest

TS received lecture honoraria from Daiichi Sankyo Co. LTD. and unlimited research grants from Novartis Takeda, Fujifilm-Wako and Daiichi Sankyo Co. LTD. Esaxerenone was provided by Daiichi Sankyo Co. LTD.

The remaining authors declare that the research was conducted in the absence of any commercial or financial relationships that could be construed as a potential conflict of interest.

## Publisher’s note

All claims expressed in this article are solely those of the authors and do not necessarily represent those of their affiliated organizations, or those of the publisher, the editors and the reviewers. Any product that may be evaluated in this article, or claim that may be made by its manufacturer, is not guaranteed or endorsed by the publisher.

## Supplementary material

Supplementary Figure 1 ESA inhibited aldosterone-induced cell proliferation in mice. Immunohistochemical staining using antibodies against Ki-67 to examine cell proliferation (n=6). The data are presented as the mean ± SD, *p < 0.05 vs. CON, #p < 0.05 vs. ALD.

Supplementary Figure 2 Macrophage proliferation in the kidneys of UNX + ALD-infused mice. Immunofluorescent staining of kidney sections with antibodies against the macrophage marker F4/80 (FITC, green) and the proliferating cell nuclear antigen marker Ki-67 (TRITC, red) to identify proliferating macrophages, and nuclei were stained with DAPI in blue.

Supplementary Figure 3 The reduction of serum K^+^ level in UNX + ALD-infused mice. Serum K^+^ level was measured by protein hydrolysis enzyme method. The data are presented as the mean ± SD, *p < 0.05 vs. CON, #p < 0.05 vs. ALD.

## References

[1] Xie Y Bowe B Mokdad AH Xian H Yan Y Li T. Analysis of the global burden of disease study highlights the global, regional, and national trends of chronic kidney disease epidemiology from 1990 to 2016. Kidney Int (2018) 94:567–81. doi: 10.1016/j.kint.2018.04.011 30078514

[2] Webster AC Nagler EV Morton RL Masson P. Chronic kidney disease. Lancet (2017) 389:1238–52. doi: 10.1016/S0140-6736(16)32064-5 27887750

[3] Falke LL Gholizadeh S Goldschmeding R Kok RJ Nguyen TQ. Diverse origins of the myofibroblast-implications for kidney fibrosis. Nat Rev Nephrol (2015) 11:233–44. doi: 10.1038/nrneph.2014.246 25584804

[4] Klingberg F Hinz B White ES. The myofibroblast matrix: Implications for tissue repair andfibrosis. J Pathol (2013) 229:298–309. doi: 10.1002/path.4104 PMC4005341 22996908

[5] Piera-Velazquez S LI Z Jimenez SA. Role of endothelial-mesenchymal transition (EndoMT) in the pathogenesis of fibrotic disorders. Am J Pathol (2011) 179:1074–80. doi: 10.1016/j.ajpath.2011.06.001 PMC3157273 21763673

[6] Shuang W Xiao-Ming M Yee-Yung N Ma FY Shuang Z Yang Z. TGF-β/Smad3 signalling regulates the transition of bone marrowderived macrophages into myofibroblasts during tissue fibrosis. Oncotarget (2016) 7:8809–22. doi: 10.18632/oncotarget.6604 PMC4891006 26684242

[7] Gao X Yamazaki Y Tezuka Y Omata K Ono Y. Pathology of aldosterone biosynthesis and its action. Tohoku J Exp Med (2021) 254:1–15. doi: 10.1620/tjem.254.1 34011803

[8] Fourkiotis V Hanslik G Hanusch F Lepenies J Quinkler M. Aldosterone and the kidney. Hormone Metab Res (2011) 44:194–201. doi: 10.1055/s-0031-1295461 22161301

[9] Xiong Y Chang Y Hao J Zhang C Yang F Wang Z. Eplerenone attenuates fibrosis in the contralateral kidney of UUO rats by preventing macrophage-to-Myofibroblast transition. Front Pharmacol (2021) 12. doi: 10.3389/fphar.2021.620433 PMC7943730 33716747

[10] Wang C-H Wang Z Liang L-J Wang X-T Ma X-L Liu B-B. The inhibitory effect of eplerenone on cell proliferation in the contralateral kidneys of rats with unilateral ureteral obstruction. Nephron (2017) 136:328–38. doi: 10.1159/000473702 28402979

[11] Arai K Homma T Morikawa Y Ubukata N Tsuruoka H Aoki K. Pharmacological profile of CS-3150, a novel, highly potent and selective non-steroidal mineralocorticoid receptor antagonist. Eur J Pharmacol (2015) 761:226–34. doi: 10.1016/j.ejphar.2015.06.015 26073023

[12] Arai K Tsuruoka H Homma T. CS-3150, a novel non-steroidal mineralocorticoid receptor antagonist, prevents hypertension and cardiorenal injury in Dahl salt-sensitive hypertensive rats. Eur J Pharmacol (2015) 769:266–73. doi: 10.1016/j.ejphar.2015.11.028 26607463

[13] Ma X Chang Y Xiong Y Wang Z Wang X Xu Q. Eplerenone ameliorates cell pyroptosis in contralateral kidneys of rats with unilateral ureteral obstruction. Nephron (2019) 142:233–42. doi: 10.1159/000497489 30799394

[14] Acelajado MC Pimenta E Calhoun DA. Salt and aldosterone: A concert of bad effects. Hypertension (2010) 56:804–5. doi: 10.1161/HYPERTENSIONAHA.110.160960 20921423

[15] Kawarazaki W Nagase M Yoshida S Takeuchi M Ishizawa K Ayuzawa N. Angiotensin II- and salt-induced kidney injury through Rac1-mediated mineralocorticoid receptor activation. J Am Soc Nephrol (2012) 23:997–1007. doi: 10.1681/ASN.2011070734 22440899 PMC3358757

[16] Ferreira NS Tostes RC Paradis P Schiffrin EL. Aldosterone, inflammation, immune system, and hypertension. Am J Hypertens (2021) 34:15–27. doi: 10.1093/ajh/hpaa137 32820797 PMC7891246

[17] Spencer S Wheeler-Jones C Elliott J. Aldosterone and the mineralocorticoid receptor in renal injury: A potential therapeutic target in feline chronic kidney disease. J Vet Pharmacol Ther (2020) 43:243–67. doi: 10.1111/jvp.12848 PMC8614124 32128854

[18] Han HI Skvarca LB Espiritu EB Davidson AJ Hukriede NA. The role of macrophages during acute kidney injury: Destruction and repair. Pediatr Nephrol (2019) 34:561–9. doi: 10.1007/s00467-017-3883-1 PMC6066473 29383444

[19] Deshmane SL Kremlev S Amini S Sawaya BE. Monocyte chemoattractant protein-1 (MCP-1): An overview. J Interferon Cytokine Res (2009) 29:313–26. doi: 10.1089/jir.2008.0027 PMC2755091 19441883

[20] Jenkins SJ Ruckerl D Cook PC Jones LH Finkelman FD Rooijen NV. Local macrophage proliferation, rather than recruitment from the blood, is a signature of TH2 inflammation. Science (2011) 332:1284–8. doi: 10.1126/science.1204351 PMC3128495 21566158

[21] Gerlach BD Ampomah PB Yurdagul A Liu C Lauring MC Wang X. Efferocytosis induces macrophage proliferation to help resolve tissue injury. Cell Metab (2021) 33:2445–2463.e8. doi: 10.1016/j.cmet.2021.10.015 34784501 PMC8665147

[22] Fidler TP Xue C Yalcinkaya M Hardaway B Abramowicz S Xiao T. The AIM2 inflammasome exacerbates atherosclerosis in clonal haematopoiesis. Nature (2021) 592:296–301. doi: 10.1038/s41586-021-03341-5 33731931 PMC8038646

[23] Zhang H Xu A Sun X Yang Y Zhang L Bai H. Self-maintenance of cardiac resident reparative macrophages attenuates doxorubicin-induced cardiomyopathy through the SR-A1-c-Myc axis. Circ Res (2020) 127:610–27. doi: 10.1161/CIRCRESAHA.119.316428 32466726

[24] Eardley KS Cockwell P. Macrophages and progressive tubulointerstitial disease. Kidney Int (2005) 68:437–55. doi: 10.1111/j.1523-1755.2005.00422.x 16014021

[25] Yonemoto S Machiguchi T Nomura K Minakata T Nanno M Yoshida H. Correlations of tissue macrophages and cytoskeletal protein expression with renal fibrosis in patients with diabetes mellitus. Clin Exp Nephrol (2006) 10:186–92. doi: 10.1007/s10157-006-0426-7 17009076

[26] Vernon MA Mylonas KJ Hughes J. Macrophages and renal fibrosis. Semin Nephrol (2010) 30:302–17. doi: 10.1016/j.semnephrol.2010.03.004 20620674

[27] Li C Ding XY Xiang DM Xu J Huang XL Hou FF. Enhanced M1 and impaired M2 macrophage polarization and reduced mitochondrial biogenesis *via* inhibition of AMP kinase in chronic kidney disease. Cell Physiol Biochem (2015) 36:358–72. doi: 10.1159/000430106 25967974

[28] Floege J Eitner F Alpers CE. A new look at platelet-derived growth factor in renal disease. J Am Soc Nephrol (2008) 19:12–23. doi: 10.1681/ASN.2007050532 18077793

[29] Henderson NC Mackinnon AC Farnworth SL Kipari T Haslett C Iredale JP. Galectin-3 expression and secretion links macrophages to the promotion of renal fibrosis. Am J Pathol (2008) 172:288–98. doi: 10.2353/ajpath.2008.070726 PMC2312353 18202187

[30] Yona S Kim K-W Wolf Y Mildner A Varol D Breker M. Fate mapping reveals origins and dynamics of monocytes and tissue macrophages under homeostasis. Immunity (2013) 38:79–91. doi: 10.1016/j.immuni.2012.12.001 23273845 PMC3908543

[31] Zhang CJ Li H Xiong YZ Chang Y Yang F Ma XL. Chronic intermittent hypoxia induces renal fibrosis through MR activation. Exp Gerontol (2022) 163:111780. doi: 10.1016/j.exger.2022.111780 35346763

[32] Martin-Fernandez B Rubio-Navarro A Cortegano I Ballesteros S Alia M Cannata-Ortiz P. Aldosterone induces renal fibrosis and inflammatory M1-macrophage subtype *via* mineralocorticoid receptor in rats. PloS One (2016) 11:e0145946. doi: 10.1371/journal.pone.0145946 26730742 PMC4701403

[33] Bienvenu LA Morgan J Rickard AJ Tesch GH Cranston GA Fletcher EK. Macrophage mineralocorticoid receptor signaling plays a key role in aldosterone-independent cardiac fibrosis. Endocrinology (2012) 153:3416–25. doi: 10.1210/en.2011-2098 22653557

[34] Usher MG Duan SZ Ivaschenko CY Frieler RA Berger S Schütz G. Myeloid mineralocorticoid receptor controls macrophage polarization and cardiovascular hypertrophy and remodeling in mice. J Clin Invest (2010) 120:3350–64. doi: 10.1172/JCI41080 PMC2929712 20697155

[35] Han JS Choi BS Yang CW Kim YS. Aldosterone-induced TGF-beta1 expression is regulated by mitogen-activated protein kinases and activator protein-1 in mesangial cells. J Korean Med Sci (2009) 24 Suppl:S195–203. doi: 10.3346/jkms.2009.24.S1.S195 PMC2633178 19194552

[36] Humphreys BD Lin SL Kobayashi A Hudson TE Nowlin BT Bonventre JV. Fate tracing reveals the pericyte and not epithelial origin of myofibroblasts in kidney fibrosis. Am J Pathol (2010) 176:85–97. doi: 10.2353/ajpath.2010.090517 20008127 PMC2797872

[37] Qi W Chen X Polhill TS Sumual S Twigg S Gilbert RE. TGF-beta1 induces IL-8 and MCP-1 through a connective tissue growth factor-independent pathway. Am J Physiol Renal Physiol (2006) 290:F703–9. doi: 10.1152/ajprenal.00254.2005 16204411

[38] Sun Y Zhang J Zhang JQ Ramires F. Local angiotensin II and transforming growth factor-?1 in renal fibrosis of rats. Hypertension (2000) 35:1078–84. doi: 10.1161/01.HYP.35.5.1078 10818068

[39] Liu L Guo H Song A Huang J Yang P. Progranulin inhibits LPS-induced macrophage M1 polarization *via* NF-кB and MAPK pathways. BMC Immunol (2020) 21:32. doi: 10.1186/s12865-020-00355-y PMC7275413 32503416

[40] Islam SU Lee JH Shehzad A Ahn EM Lee YS. Decursinol angelate inhibits LPS-induced macrophage polarization through modulation of the NFκB and MAPK signaling pathways. Molecules (2018) 23:1880. doi: 10.3390/molecules23081880 PMC6222640 30060484

[41] Ray PE Suga SI Liu XH Huang X Johnson RJ. Chronic potassium depletion induces renal injury, salt sensitivity, and hypertension in young rats. Kidney Int (2001) 59:1850–8. doi: 10.1046/j.1523-1755.2001.0590051850.x 11318956

[42] Pham TD Verlander JW Wang Y Romero CA Yue Q Chen C. Aldosterone regulates pendrin and epithelial sodium channel activity through intercalated cell mineralocorticoid receptor-dependent and -independent mechanisms over a wide range in serum potassium. J Am Soc Nephrol (2020) 31:483–99. doi: 10.1681/ASN.2019050551 PMC7062219 32054691

[43] Zhao L Sun LN Nie HB Wang XL Guan GJ. Berberine improves kidney function in diabetic mice *via* AMPK activation. PloS One (2014) 9:e113398. doi: 10.1371/journal.pone.0113398 25409232 PMC4237447

