# Supplementary figures and images for: Esaxerenone inhibits the macrophage-to-myofibroblast transition through mineralocorticoid receptor/TGF-β1 pathway in mice induced with aldosterone
Source: Front Immunol. 2022 Sep 6;13:948658. doi: 10.3389/fimmu.2022.948658 (PMC9485811; doi:10.3389/fimmu.2022.948658)

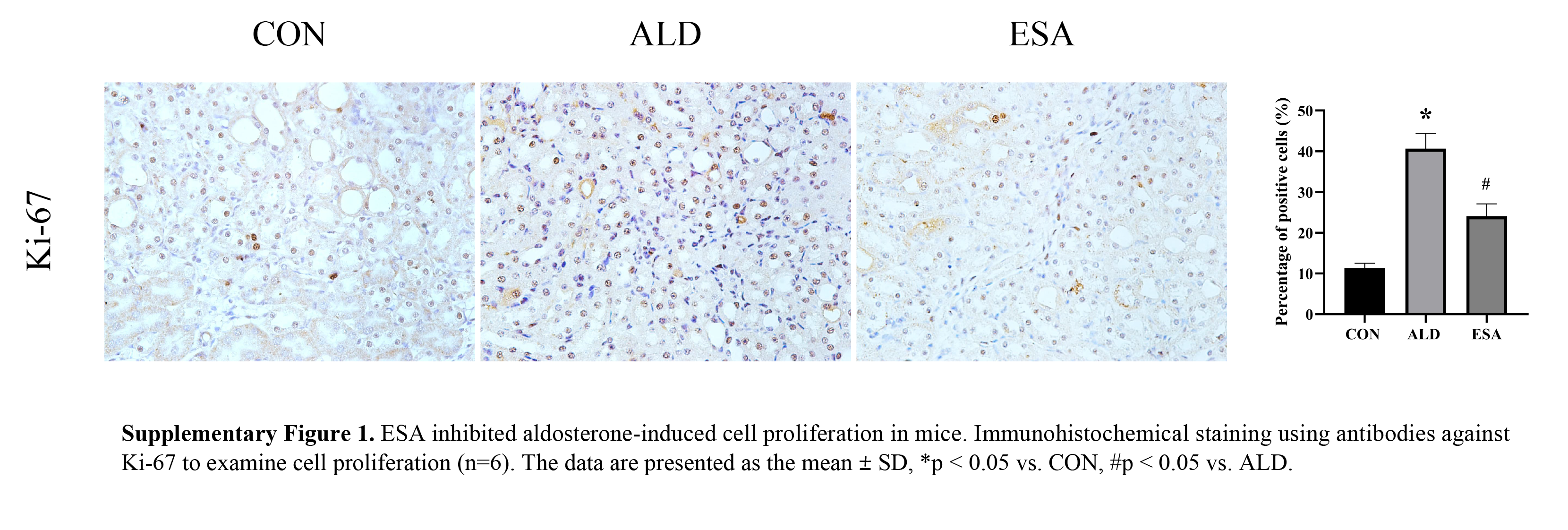

Supplement: Supplementary Figure 1 — ESA inhibited aldosterone-induced cell proliferation in mice. Immunohistochemical staining using antibodies against Ki-67 to examine cell proliferation (n=6). The data are presented as the mean ± SD, *p < 0.05 vs. CON, #p < 0.05 vs. ALD. [file Image_1.tif]

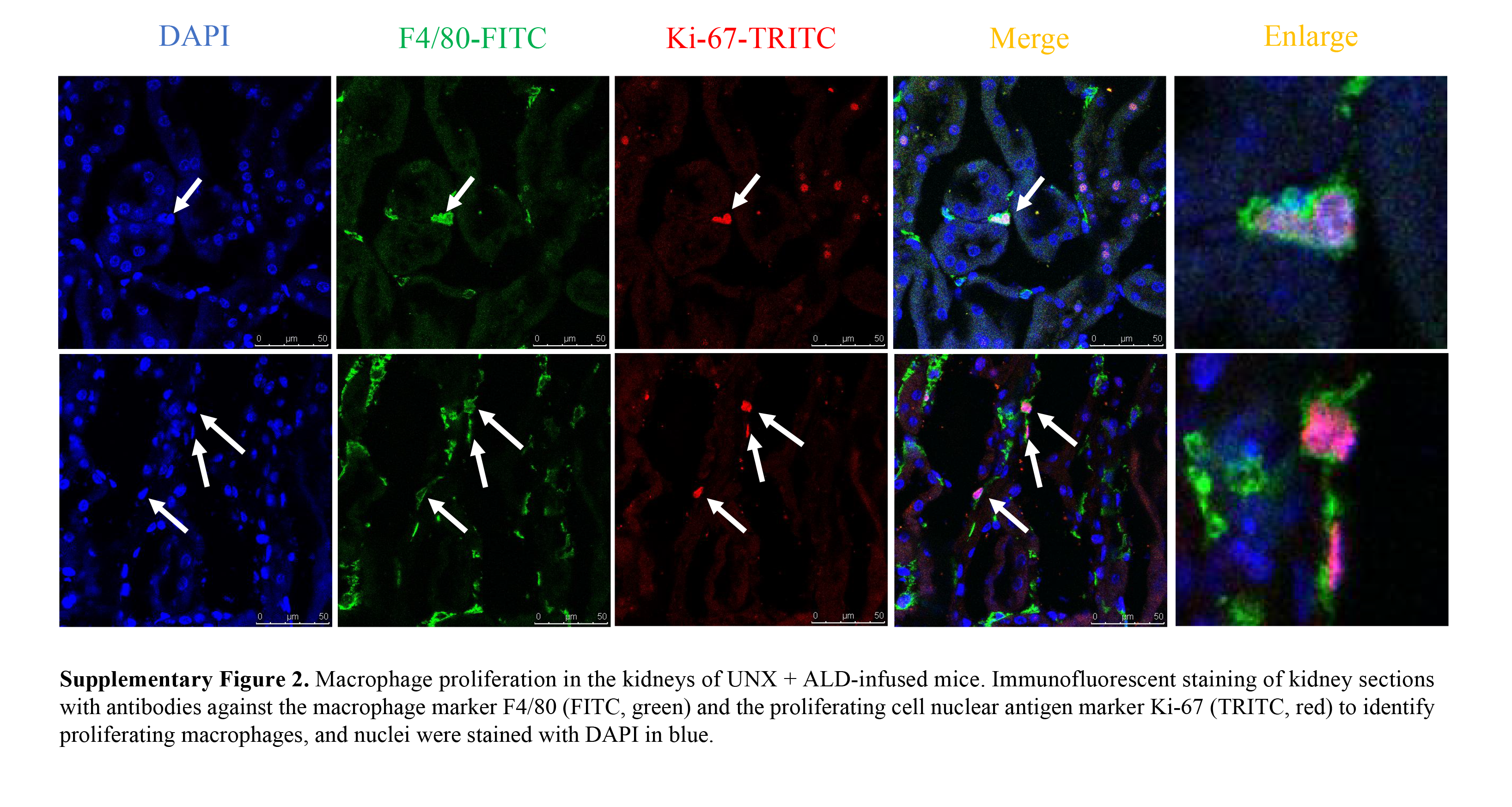

Supplement: Supplementary Figure 2 — Macrophage proliferation in the kidneys of UNX + ALD-infused mice. Immunofluorescent staining of kidney sections with antibodies against the macrophage marker F4/80 (FITC, green) and the proliferating cell nuclear antigen marker Ki-67 (TRITC, red) to identify proliferating macrophages, and nuclei were stained with DAPI in blue. [file Image_2.tif]

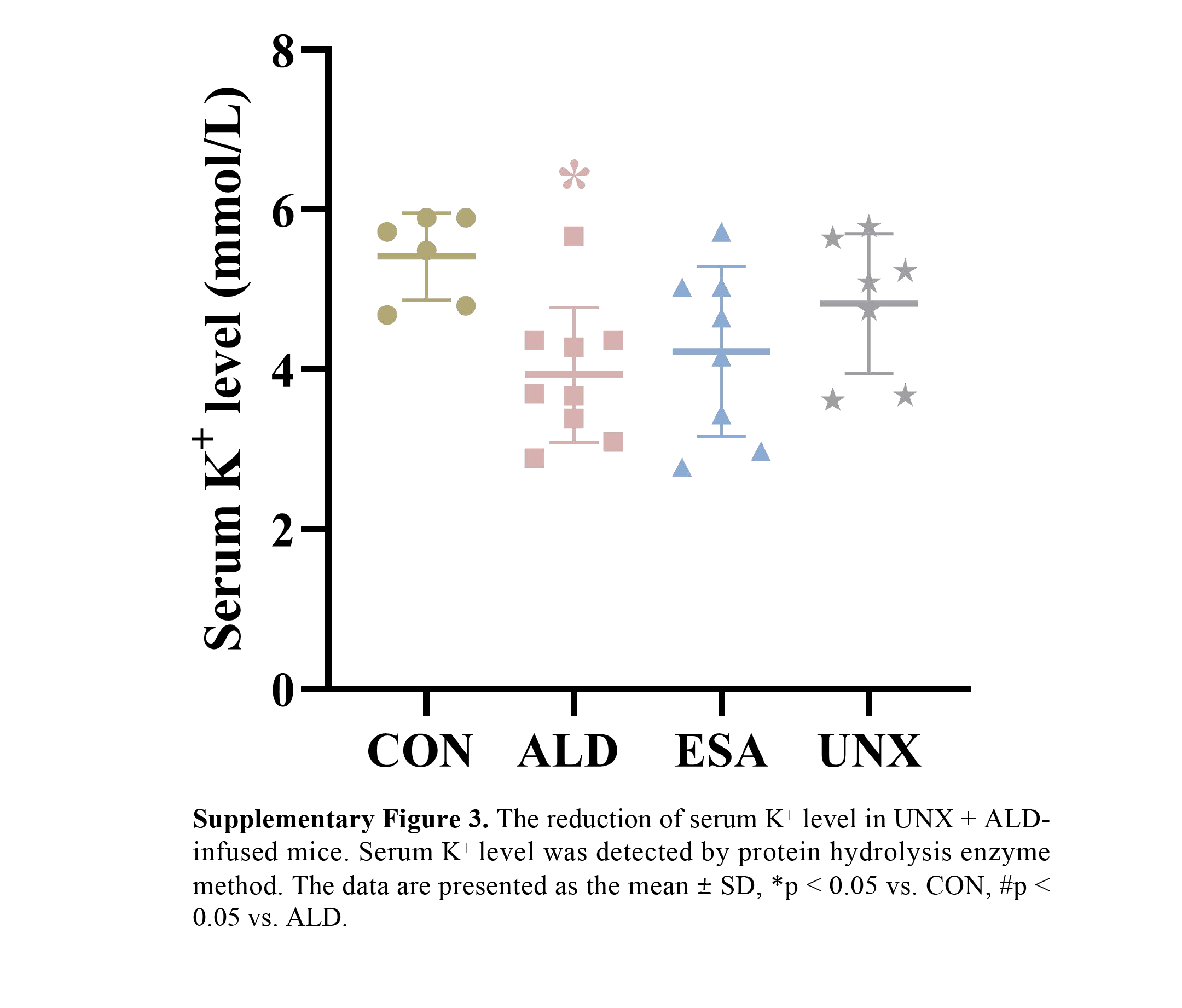

Supplement: Supplementary Figure 3 — The reduction of serum K+ level in UNX + ALD-infused mice. Serum K+ level was measured by protein hydrolysis enzyme method. The data are presented as the mean ± SD, *p < 0.05 vs. CON, #p < 0.05 vs. ALD. [file Image_3.tif]
